# Infant and adult visual attention during an imitation demonstration

**DOI:** 10.1002/dev.21147

**Published:** 2013-09-04

**Authors:** Gemma Taylor, Jane S Herbert

**Affiliations:** Department of Psychology, University of SheffieldWestern Bank, Sheffield, S10 2TN, UK

**Keywords:** infant, learning, attention, memory

## Abstract

Deferred imitation tasks have shown that manipulations at encoding can enhance infant learning and memory performance within an age, suggesting that brain maturation alone cannot fully account for all developmental changes in early memory abilities. The present study investigated whether changes in the focus of attention during learning might contribute to improving memory abilities during infancy. Infants aged 6, 9, and 12 months, and an adult comparison group, watched a video of a puppet imitation demonstration while visual behavior was recorded on an eye tracker. Overall, infants spent less time attending to the video than adults, and distributed their gaze more equally across the demonstrator and puppet stimulus. In contrast, adults directed their gaze primarily to the puppet. When infants were tested for their behavioral recall of the target actions, “imitators” were shown to have increased attention to the person and decreased attention to the background compared to “non-imitators.” These results suggest that attention during learning is related to memory outcome and that changes in attention may be one mechanism by which manipulations to the learning event may enhance infant recall memory. © 2013 The Authors. Developmental Psychobiology published by Wiley Periodicals, Inc.

## Introduction

Although very young infants can encode, store and retrieve information, there are considerable developmental changes in learning and memory abilities across the infancy period (for reviews, see Hayne, 2004; Jones & Herbert, 2006). Across paradigms, older infants have been shown to encode information faster (e.g., Barr, Dowden, & Hayne, 1996; Hill, Borovsky, & Rovee-Collier, 1988; Rose, 1983), retain information over a longer duration (e.g., Barr & Hayne, 2000; Herbert, Gross, & Hayne, 2006; Morgan & Hayne, 2006) and reproduce longer multi-step sequences from memory (Barr et al., 1996; Kressley-Mba, Lurg, & Knopf, 2005) than younger infants. During this time, there are also considerable developments in infants’ ability to use their knowledge flexibly. With age, infants are increasingly able to learn behaviors from indirect sources, such as books or television (for review, see Barr, 2010), to retrieve their memories in new contexts (e.g., Borovsky & Rovee-Collier, 1990; Hayne, Boniface, & Barr, 2000; Jones, Pascalis, Eacott, & Herbert, 2011; Learmonth, Lamberth, & Rovee-Collier, 2004), and to apply their knowledge when confronted with stimuli that are similar but not identical to those that were present during learning (e.g., Fagen & Rovee, 1976; Hayne, MacDonald, & Barr, 1997; Jones & Herbert, 2008). Although developmental changes in early learning and memory are well documented, considerably less is known about the mechanisms underpinning these changes.

Explanations for developmental changes in learning and memory have frequently focused on the development of the medial temporal lobe (for reviews, see Nelson, 1995,2000; Richmond & Nelson, 2007). For example, Nelson (1995, 2000) has marked the functional maturation of the hippocampal circuitry such as the dendate gyrus, which begins between 6 and 12 months of age, as the time at which early forms of learning and memory start to become adult-like. However, a simple biological explanation for memory development fails to fully account for the growing body of research showing that by manipulating the learning session, infant memory performance can be enhanced. One paradigm in particular, deferred imitation, has revealed the extent to which the duration and flexibility of infant memory is dependent upon the experiences provided during learning.

In the deferred imitation paradigm, infants observe an experimenter demonstrating a series of actions with a novel object and the infant’s ability to reproduce those actions is assessed either immediately or after a delay (for review, see Hayne, 2004). Providing 6-month-old infants with six rather than three demonstrations of a three-step sequence of target actions on a puppet (removing, shaking, and replacing the puppet’s mitten) facilitates their ability to reproduce the actions after a 24-hr delay, consistent with 12-month-olds who only receive three demonstrations (Barr et al., 1996). Similarly, although infant memory retrieval is highly dependent upon an exact match between the conditions at encoding and retrieval (for review, see Jones & Herbert, 2006) providing infants with additional language cues during the demonstration and test (Herbert, 2011), the opportunity to practice the target actions following the demonstration (Learmonth et al., 2004) or additional experience with multiple stimulus exemplars (Barr, Marrott, & Rovee-Collier, 2003) can, however, facilitate infant’s ability to retrieve their memories when tested with stimuli that differ from those present at encoding. Taken together, these findings reveal that infants’ learning and memory capabilities can be enhanced within an age. Thus, it is unlikely that brain maturation can solely account for changes in learning and memory during the first year of life.

It has recently been proposed that developmental changes in learning and memory may, in part, be attributed to changes in attention across age (Rovee-Collier & Giles, 2010). That is, manipulations to the amount of learning, or to the cues available during learning, may enhance infant memory by altering what or how infants attend to the events. To investigate changes in attention, researchers typically measure looking behavior during stimulus presentation. Using eye tracker methodology, age-related changes in distractibility and the features that attract attention have been documented during the infancy period. Specifically, 3-month-old infants are slower to orient to a target following a distracter than 6-month-old infants, who are in turn, slower than 9-month-old infants (Amso & Johnson, 2008). In addition, when attending to a complex cartoon clip, 3-month-old infants scan the whole visual scene indiscriminately, while 6-month-old infants focus gaze on salient items in the scene (e.g., brightly colored or moving items) and 9-month-old infants focus gaze primarily on faces (Frank, Vul, & Johnson, 2009). Thus, differences have been observed in the way infants of different ages attend to the same event.

Recently, studies have started to address whether changes in attention reflect changes in the way that infants understand events. In Gredebäck, Stasiewicz, Falck-Ytter, Rosander, and von Hofsten (2009), 10- and 14-month-old infants’ visual behavior was tracked while they attended to a video of a person moving objects across a table and into a container. Ten-month-old infants tracked the actions reactively; that is, their gaze tracked the adult’s hand as it moved across the table and reached the goal. In contrast, 14-month-old infants made predictive gaze shifts, shifting to the goal of the reaching and containment actions prior to the adult’s hand reaching the object or container. In a related study, Cannon, Woodward, Gredebäck, von Hofsten, and Turek (2012) showed 12-month-old infants a video of a person placing three balls into a bucket either before or after the infant had engaged in containment activities themselves. Overall, there was a positive relationship between containment action production prior to viewing the video and infants’ subsequent tendency to make predictive gaze shifts during the video. Age- and experience-related progression toward predictive looking behavior might, therefore, reflect infants’ increasing understanding about the intentions and goals of another’s actions.

Although research has considered how attention changes during infancy, less is known about the extent to which these changes impact on subsequent learning and memory. One notable exception is an eye tracking study by Vivanti, Nadig, Ozonoff, and Rogers (2008) conducted with older children (aged 8–15 years) who were typically developing or diagnosed with autism. In this study, children watched video demonstrations of meaningful actions performed with an object (i.e., flattening dough with a rolling pin), and non-meaningful gestures (i.e., arm flexing at the elbow), and were then given the opportunity to imitate the demonstrator’s behaviors. Both groups of children attended to the action region for a similar amount of time and increased attention to the demonstrator’s face when non-meaningful gestures were presented. However, children with autism spent 7% (meaningful actions) to 13% (non-meaningful gestures) less time attending to the demonstrator and showed less imitation precision than typically developing children. These results are consistent with previous findings indicating that successful imitation by young children and children with autism is linked with attention to the person’s face (Carpenter, Tomasello, & Savage-Rumbaugh, 1995; Williams, Whiten, & Singh, 2004). However, Vivanti et al. (2008) also found that it was attention to the action region during the demonstration of non-meaningful gestures that was correlated to the imitation precision of those actions. Thus, this study suggests a potential link between the focus of attention during an imitation demonstration and memory outcome, at least in older children.

During infancy, the relationship between attention and memory outcome is less clear, and may be affected by the type of stimuli used during familiarization. When 3- (Bronson, 1991) and 5-month-old infants (Jankowski, Rose, & Feldman, 2001) are familiarized with a static picture, visual recognition for that stimulus is related to short, distributed looking behavior. In contrast, using a video of the well-established puppet imitation task (see also Barr et al., 1996; Barr, Muentener, & Garcia, 2007), Taylor and Herbert (2013) found limited evidence for a relationship between attention during learning, measured using an eye tracker, and visual recognition memory at 6 and 9 months of age. Although imitation studies using the puppet task traditionally involve the experimenter using empty language cues to direct infant’s attention to the target event, the video demonstration in this study was silent, to be consistent with previous eye tracking studies (e.g., Vivanti et al., 2008). Overall, infants spent approximately 30% of their viewing time attending to the person and the puppet, and approximately 12% of the time attending to the background (for the remainder of the time, infants were not attending to the video). Despite attending to the target features on the video for some of their viewing time, both 6- and 9-month-old infants showed only limited evidence of recognition memory when tested immediately with static photographs of these features. Specifically, although infants showed evidence of recognition for the person from the video, there was no evidence of recognition for the puppet or background from the video.

Although the study by Taylor and Herbert (2013) has started to address the potential role of attention on developmental changes in infant learning and memory, the conclusions that can be drawn from this study are limited in two key ways. First, the performance of only two age groups was considered, and they were just 3 months apart in age. A particular strength of the puppet imitation task is that it can be used to examine developmental changes in learning and memory with infants from 6 to 24 months of age (for review, see Hayne, 2004). The developmental changes in memory observed on this task are relatively small between 6 and 9 months (e.g., Herbert et al., 2006; Learmonth et al., 2004) compared to developmental changes between 6 and 12 months (e.g., Barr et al., 1996; Hayne et al., 1997). Furthermore, given that 9-month-old infants are on the cusp of showing increased flexibility in their memory abilities, with considerable individual differences being observed at this age (e.g., Herbert et al., 2006), it is important to assess memory performance when abilities are more robust, later during infancy, or even in adulthood. Second, the only measure of memory obtained by Taylor and Herbert (2013) was infants’ visual recognition for the components of the demonstration video. Prior research has shown that 6-month-old infants may fail to show recognition memory for the puppet following a live demonstration, even though they still exhibit behavioral recall for the target actions that were performed with it (Gross, Hayne, Herbert, & Sowerby, 2002). Thus, further research is needed to determine the relationship between attention and learning outcome, assessed by both recognition and recall memory procedures.

In the present study, infants aged 6, 9, and 12 months, and an adult comparison group watched a video of a model performing a puppet imitation task demonstration which had been filmed against a colorful background context. We hypothesized that eye tracking data would show participants’ focus of attention to the person, puppet, and background details change across age (see Amso & Johnson, 2008; Frank et al., 2009; Taylor & Herbert, 2013). Consistent with Taylor and Herbert (2013), we hypothesized that 6- and 9-month-old infants would attend to the person and puppet for similar amounts of time, while still giving some visual attention to the background. Theoretically, increased attention to the puppet should facilitate infants’ ability to imitate the target actions by enhancing the effectiveness of the puppet as a retrieval cue and decreasing the importance of more peripheral details, like the person and the background (for similar argument, see Taylor & Herbert, 2013). Therefore, we hypothesized that by 12 months of age, infants would show increased focus on the puppet and decreased focus on the background, reflecting increased memory flexibility at this age, and perhaps being more consistent with our adult participants.

To assess learning and memory outcome, the present study included a visual recognition test for infants’ and adults’ memory for the person, puppet and background, as well as a behavioral recall test for infants’ memory for the demonstrated target actions. For the visual recognition test, a control group of participants were also tested to determine whether participants showed a spontaneous preference for the “familiar” stimuli image when both images were novel. Consistent with Taylor and Herbert (2013), we hypothesized that younger infants would show only limited evidence of recognition memory, possibly due to the use of complex moving social stimuli during familiarization (also see Brown, Robinson, Herbert, & Pascalis, 2006). In contrast, we hypothesized that adults, and potentially older infants, would recognize all three components from the video, given that complex dynamic stimuli are typically used for adult visual recognition tasks (e.g., Richmond, Colombo, & Hayne, 2007; Richmond, Sowerby, Colombo, & Hayne, 2004). Given that behavioral recall for the target actions demonstrated in the puppet imitation task is not an age-appropriate memory measure for adults, this aspect was only conducted with infants. We hypothesized that infants would show increasing levels of behavioral recall between 6 and 12 months of age, reflecting their increasing ability to learn and recall the target actions from both live and televised demonstrations (see Barr et al., 1996; 2007). Infant imitation performance was compared to a control group of infants who had not seen the target actions demonstrated.

Finally, the present study also considered whether changes in visual attention during learning relates to subsequent learning outcome. In adults and older children, there is evidence that looking during an action demonstration is related to subsequent recall (Hard, Recchia, & Tversky, 2011; Vivanti et al., 2008). Thus, we hypothesized that infants who exhibited behavioral recall would also show increased attention to the puppet and decreased attention to the background while viewing the demonstration. Therefore, the overarching aim of the present study is to determine whether a change in attention towards the central stimulus, and away from background details, is central to the gradual development of retention and memory flexibility during infancy.

## Methods

### Participants

The final sample consisted of 32 six-month-old infants (19 males, 13 females), 32 nine-month-old infants (18 males, 14 females), 32 twelve-month-old infants (18 males, 14 females), and 32 undergraduate psychology students (7 males, 25 females, *M* = 19.13 years, *SD* = 1.48). Participants were randomly assigned to an experimental or control condition (*n* = 16 of each age group in each condition). Infants were typically developing and were tested within 10 days of their 6-, 9-, and 12-month birthday. An additional 56 infants (32 six-month-olds, 16 nine-month-olds, and 8 twelve-month-olds) were tested but excluded due to: calibration failure (*n* = 18), looking at the video for less than 5 s (*n* = 17), exhibiting positional biases on more than one of the recognition tests (*n* = 13) and fussiness during the experiment (*n* = 8). Calibration failure for the infant participants was primarily due to movement during calibration and not looking for long enough at each calibration point. Eighteen additional adults were also tested but excluded due to calibration failure as the result of eye makeup or bright blue irises which made pupil detection difficult. This overall attrition rate of 36.6% is consistent with previous studies employing a similar methodology (38% Taylor & Herbert, 2013; 31% Amso & Johnson, 2008). The study was approved by the Department of Psychology Ethics Sub-Committee at the University of Sheffield.

### Stimuli

**Figure 1:**
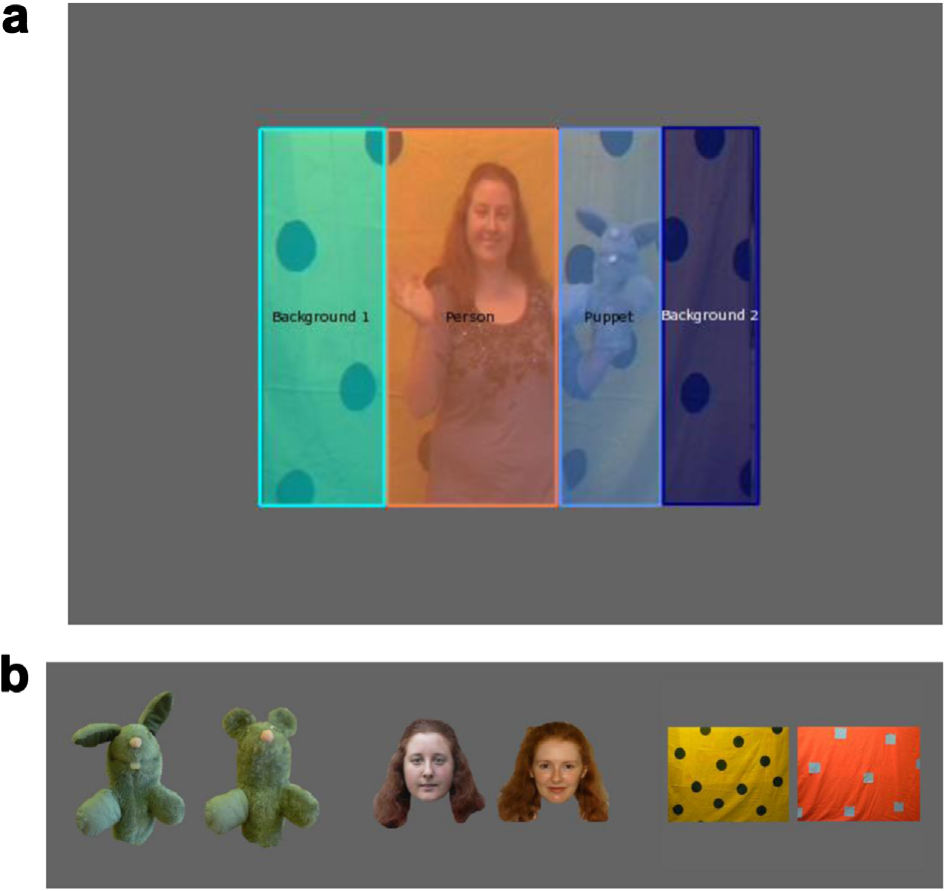
Screenshot of the (a) experimental video with the AOIs: background, puppet, and person; and (b) recognition tests for the person, puppet, and background.

Two videos were created for use during the learning phase. The experimental video (75 s in duration) featured a female adult demonstrating a series of target actions on a gray rabbit hand puppet while standing in front of a distinctive background (see Fig. 1a). The puppet (27 cm × 30 cm) featuring on the video had a matching gray mitten placed over its right hand (8 cm × 9 cm) which could be removed (see Barr et al., 1996). The same puppet was presented during the behavioral recall session. The yellow and green polka dot background was made from material and adapted from that used by Meltzoff and colleagues (Barnat, Klein, & Meltzoff, 1996; Klein & Meltzoff, 1999). This background functioned as a distinctive feature for use in the recognition memory test.

At the beginning of the experimental video, the demonstrator waved hello, tapped the puppet’s mitten to ring the bell inside before removing the mitten (step 1), shaking the mitten three times to ring the bell inside (step 2), and replacing the mitten (step 3). The target actions were repeated six times in succession (*M* for each complete demonstration = 10.87 s, range = 10.32–11.60 s), after which the experimenter waved goodbye. All participants saw six demonstrations of the target actions, consistent with Barr et al. (2007) who found that following six video demonstrations, 6- and 12-month-old infants perform at the same level as age-matched infants who saw the actions demonstrated live.

The control video (98 s in duration) was designed to provide participants in the control condition with a similar, but unrelated viewing experience. The video featured an imitation demonstration, but all features were different from the experimental video: a second female model demonstrated a sequence of actions with an unrelated rattle stimulus (see Herbert & Hayne, 2000) while standing against a plain pale green background.

In both videos, the model maintained a positive expression throughout the demonstrations but did not provide any verbal cues during the presentation, consistent with Taylor and Herbert (2013). Naturally occurring sound effects were, however, audible (e.g., the jingle bell in the mitten) to encourage infant attention to the screen. Each video was presented in the center of the screen at a size of approximately 20.8° (width) × 13.3° (height) visual angle on a uniform gray background.

A digital photograph of the entire puppet (15.2° × 18.0°), the demonstrator’s face (15.2° × 13.3°) and the room background (16.6° × 10.5°) which featured on the experimental video were used as stimuli for the recognition memory test (see Fig. 1b). Each photograph was prepared using Adobe Photoshop to adjust for individual size and cropped so that there was no extraneous information available. Each image was then paired with a related image for the recognition test (e.g., the gray rabbit puppet was paired with a gray mouse puppet) that was unfamiliar to infants in both the experimental and control conditions.

### Procedure

Participants were tested in the developmental laboratory using an SMI iView X (RED III) remote eye tracking system. Participants were seated approximately 60 cm in front of the infrared camera which was situated directly below the center of a 56 cm flat panel monitor. Each infant was seated on their caregiver’s lap and the caregiver was asked not to behaviorally or verbally direct the infant’s attention. The Experimenter tracked participants head movements on the eye tracker camera from behind a black screen. Visual fixation data was sampled from the participant’s left eye at a rate of 50 Hz with a gaze position accuracy of .5–1° using corneal reflection.

#### Visual attention during learning

To calibrate the location of the participant’s gaze, a manual calibration procedure was used. An attention-getter (an animated fish, 2.9° × 2.4°) was shown individually at five points on the screen: one at each of the corners and one in the middle of the screen. Calibration accuracy was checked and repeated as necessary. Following successful calibration, participants were presented with either the experimental or control video depending on group assignment.

#### Visual recognition test

Immediately after watching the video, a central fixation point appeared which directed the participant’s attention to the center of the screen. A visual recognition test was then presented for each item seen on the video: the puppet, person and background. Each familiar stimulus and the matched unfamiliar stimulus pairing was presented twice in succession for 5 s, with the lateral position of the images reversed on the second trial to control for potential side biases. The visual recognition test was the same duration for all participants, consistent with prior work with 6- to 18-month-old infants (Jones et al., 2011) and adults (McKee & Squire, 1993). The order in which the three stimulus types were presented was counterbalanced across participants.

#### Imitation recall test

For infants, an imitation recall test followed immediately after the visual recognition test, approximately 2–3 min after the demonstration video. Caregivers rotated their chair 180° so that their infant was facing away from the computer screen. The Experimenter then sat on the floor in front of the infant and revealed the puppet featured in the experimental video. The puppet was placed over the Experimenter’s right hand and was positioned at the infant’s eye level, within reaching distance. Infants had 90 s to reproduce the target actions. The imitation session was video recorded for later analysis.

### Data Coding

Visual attention to the video during the learning phase and the recognition memory tests was analyzed using SMI BeGaze software (SensoMotoric Instruments GmbH, Germany). In the current literature, there is no standard definition for fixations, which have been defined as anywhere between 50 and 250 ms (for discussion, see Holmqvist et al., 2011). Consistent with Taylor and Herbert (2013), fixations were defined by a minimum duration of 80 ms with a maximum dispersion of 100 pixels to allow for shorter fixations given the dynamic nature of the video. Data between fixations were defined as saccades.

#### Visual attention during learning

To investigate visual attention to features of the experimental video, three areas of interest (AOI) were defined on a screenshot of the video (see Fig. 1a). Although the video contained some movement, the person and the puppet remained in relatively the same position throughout, so static AOIs were sufficient to capture their location. The screenshot was divided lengthwise into four sections, so that in each section one of the three target features (puppet, person, and background) occupied the majority. As a result, each AOI was relatively large; puppet (84,534 square pixels which included the puppet’s face and torso), person (141,942 square pixels which included the demonstrator’s face and torso; the person’s face was not selected as a separate individual AOI due to the small size of the video on the screen and the gaze position accuracy of the eye tracker) and background (185,020 square pixels covering the remainder of the video presentation) to accommodate for any changes in calibration accuracy across the session. No correction was applied to control for the size of the AOI given that, by definition, the size of each target feature also varied accordingly.

#### Visual recognition

To assess visual recognition memory, visual attention to the novel and familiar stimulus images was calculated. For each stimulus pair, novel and familiar AOIs were defined around each image; the puppet (421,737 square pixels), person (328,032 square pixels), and background (26,590 square pixels). The proportion of time spent attending to the novel image was then calculated; novel/(novel + familiar). Not all participants contributed useable data to all three recognition tests (see Tab. 1).

**Table:** Proportion of Looking to the Novel Stimulus (±1 SD) as a Function of Participant Age and Condition

Age	Condition	Proportion of Looking to the Novel Stimulus (SD)					
		*N*	Puppet	*N*	Person	*N*	Background
6 months	Experimental	11	.48 (.13)	15	.47 (.18)	12	.44 (.24)
	Control	13	.59 (.15)	16	.46 (.19)	14	.48 (.20)
9 months	Experimental	11	.47 (.18)	14	.40* (.17)	10	.48 (.24)
	Control	13	.45 (.17)	9	.49 (.18)	6	.50 (.29)
12 months	Experimental	13	.48 (.17)	14	.50 (.14)	11	.36* (.16)
	Control	13	.44 (.21)	11	.41 (.19)	8	.30* (.13)
Adults	Experimental	16	.45 (.14)	16	.44 (.12)	16	.48 (.23)
	Control	16	.46 (.12)	16	.50 (.10)	15	.48 (.16)

* Significant at <.05.

#### Imitation recall

For the infant age groups, the videotaped imitation sessions were coded for the presence or absence of the target actions (remove, shake, replace mitten). Infants were given a score of 0–3 based on the presence or absence of each action. Not all infants contributed data for the imitation test due to failure to touch the puppet or fussiness during the test session (see Tab. 2). Higher attrition during the recall test for the 12-month-old infants reflects their unwillingness to sit still given their locomotor abilities at this age (also see Barr et al., 2007). Approximately 29% (*n* = 28) of the videos were double coded by an independent observer who was blind to experimental condition and hypotheses. Inter-observer reliability analysis was 96% (kappa = .92).

**Table 2:** Mean Imitation Score and Number of “Imitators” as a Function of Age and Condition

Age	Experimental Condition				Control Condition			
	*N*	Mean (*SD*)	Imitation	No Imitation	*N*	Mean (*SD*)	Imitation	No Imitation
6 months	16	.44 (.51)	7	9	15	.33 (.62)	4	11
9 months	16	.38 (.62)	5	11	15	.40 (.63)	5	10
12 months	12	.17 (.39)	2	10	13	.23 (.44)	3	10

## Results

### Visual Attention During Learning

Initial analyses were run on the experimental group data to establish whether overall attention to the experimental video differed as a function of age using Dwell times, the sum of all fixations and saccades. A one-way ANOVA revealed a significant difference in overall dwell time to the experimental video according to age group, *F*(3, 63) = 14.82, *p* = .000, η^2^ = .65. Overall, adults attended to the video for significantly longer (*M* = 52.39 s, *SE* = 4.95) than all infant age groups (6 months: *M* = 21.28 s, *SE* = 4.01; 9 months: *M* = 22.74 s, *SE* = 3.34; 12 months: *M* = 23.63 s, *SE* = 2.93, all *p* values = .000). Attention to the video did not differ between the infant age groups (all *p* values = 1.000).

**Figure 2:**
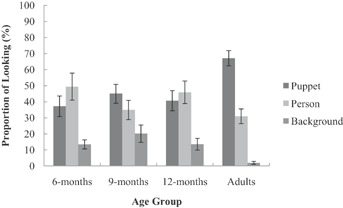
Mean looking time (±1 *SE*) to the experimental video as a proportion of the time spent looking at each AOI.

Given the difference in overall attention to the experimental video, proportion data was used to assess the overall spread of attention to each AOI (puppet, person, background) during learning. The proportion data was calculated using the total fixation time to each AOI divided by the total fixation time to the experimental video. To determine whether attention to the experimental video differed across age, a two-way (Age × AOI) mixed design ANOVA was conducted on the proportion of fixations. Given that proportion data was used, there was no main effect of age on attention to the video. However, there was a significant main effect of AOI, *F*(2, 120) = 30.129, *p* = .000, η^2^ = .55. Specifically, participants spent a greater proportion of their time attending to the puppet (*M* = 47.48%, *SE* = 2.93, *p* = .000) and person (*M* = 40.25%, *SE* = 3.34, *p* = .000) than the background (*M* = 12.28%, *SE* = 1.80). There was no difference in the proportion of time spent attending to the puppet and person (*p* = .703). There was also a significant interaction effect between age and the proportion of time spent attending to each AOI, *F*(6, 120) = 3.442, *p* = .004, η^2^ = .32 (see Fig. 2).

To further investigate the interaction between age and attention to each AOI, bonferroni post hoc analyses were conducted. Overall, adults spent a greater proportion of time (approximately 67%) attending to the puppet than the 6- (*p* = .004), 9- (*p* = .058), and 12-month-old (*p* = .013) infants, who spent between 37% and 44% of their time attending to the puppet. In contrast, adults spent less time attending to the background (attending to the background for around 2% of their time) than the infant age groups, who spent between 13% and 20% of their time attending to the background, however this finding reached significance with the 9-month-old infants only (*p* = .004; 6 months: *p* = .162; 12 months: *p* = .158). Finally, there was no difference in the proportion of time spent attending to the person (around 31–49%) and no difference in attention to the person, puppet or background between the infant age groups (all *p* values >.05).

### Visual Attention to “Off,” “Shake,” and “On” Actions

The following set of analyses assessed the spread of attention to each AOI for each action; “Off” (*M* = 2.23 s, *SD* = .13, Total = 13.36 s), “Shake” (*M* = 4.68 s, *SD* = .19, Total = 28.08 s), “On” (*M* = 3.93 s, *SD* = .31, Total = 23.60 s) during the experimental video to determine whether patterns of attention change as a function of the action being demonstrated. Given that the duration of each action differed, the total fixation time to each AOI during each action was calculated as a proportion of the total duration for that action across the six demonstrations. A three-way (Action × AOI × Age) ANOVA was run on the proportion of fixations data within each action. There was a significant main effect of action, *F*(2, 120) = 29.67, *p* = .000, η^2^ = .56. Participants spent a greater proportion of time attending to the “Shake” action (*M* = 10.14%, *SE* = .99), than the “Off” (*M* = 8.13%, *SE* = .75, *p* = .000) and “On” (*M* = 6.70%, *SE* = .75, *p* = .000) actions. Similarly, participants attended to the “Off” action for a greater proportion of time than the “On” action, *p* = .001. There was no interaction effect between action and age, *F*(6, 120) = 1.37, *p* = .233, η^2^ = .21. Thus, both the infants and adults attended predominantly to the “Shake” action, followed by attention to the “Off” and “On” actions, respectively.

**Figure 3:**
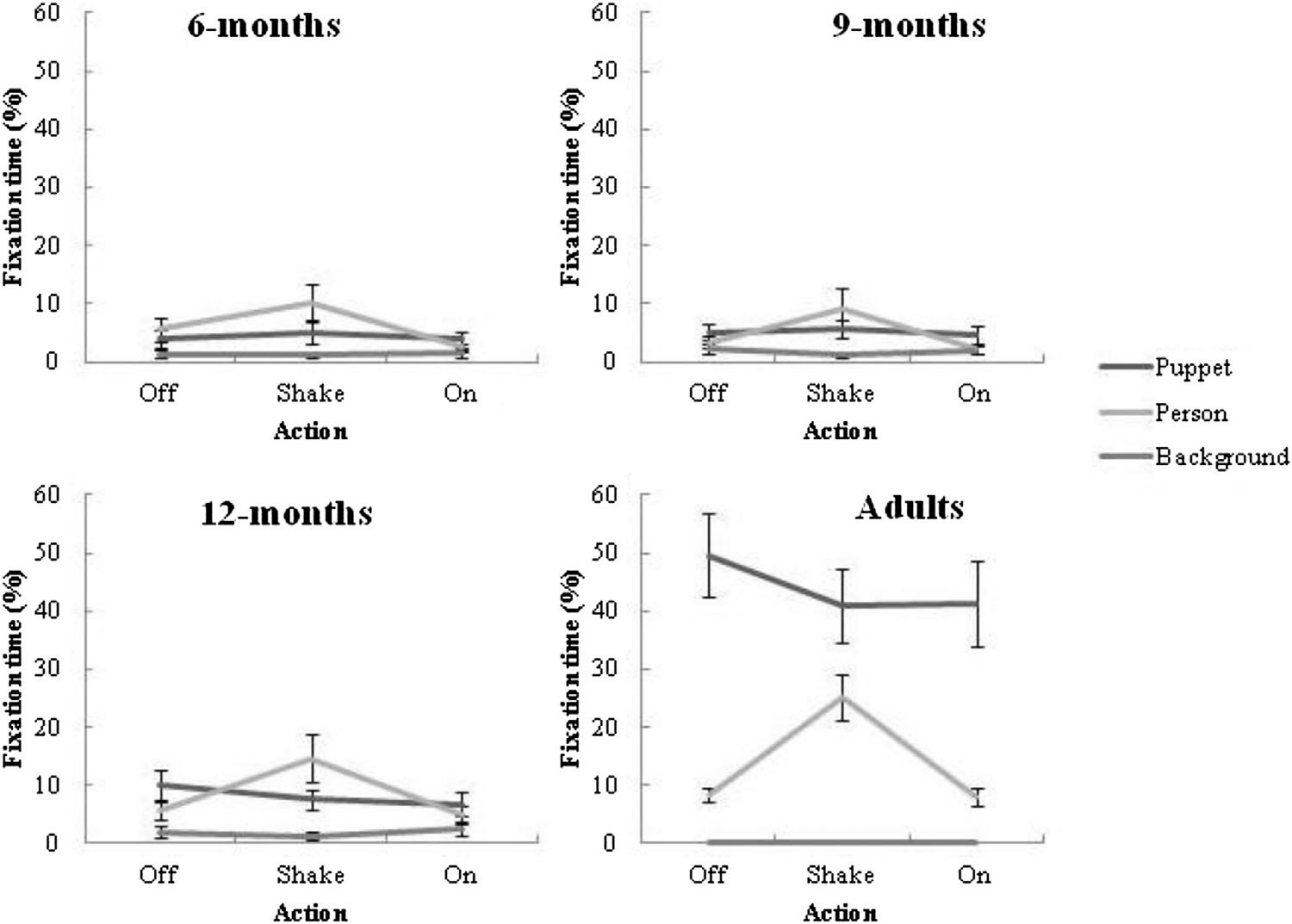
Proportion of fixations (±1 *SE*) to each AOI during the experimental video as a function of target action demonstrated and age of the participant.

The ANOVA also showed a significant AOI and action interaction, *F*(4, 240) = 23.15, *p* = .000, η^2^ = .50. Overall, attention to the puppet remained relatively stable across each action (“Off” *M* = 17.23%, *SE* = 1.98; “Shake” *M* = 14.80%, *SE* = 1.77; “On” *M* = 14.17%, *SE* = 1.97). Similarly, attention to the background remained relatively low across each action (“Off” *M* = 1.42%, *SE* = .36; “Shake” *M* = .88%, *SE* = .19; “On” *M* = 1.51%, *SE* = .41). Attention to the person however, increased for the “Shake” action (*M* = 14.75%, *SE* = 1.87) compared to the “Off” (*M* = 5.73%, *SE* = .76) and “On” actions (*M* = 4.01%, *SE* = .61). Thus, participants increased attention to the person when watching the “Shake” action. There was also a significant three-way interaction effect between action, AOI, and age group, *F*(12, 240) = 2.95, *p* = .001, η^2^ = .31 (see Fig. 3), showing that the interaction between action and AOI is different across age groups. Specifically, the infant age groups looked primarily at the person during the “Shake” action. In contrast, adults looked primarily at the puppet during each action, although attention to the person increased during the “Shake” action. Thus, adults focused on the puppet region, whilst infants focused on the person during the “Shake” action.

### Visual Recognition Memory

Next, recognition memory by the experimental groups for the puppet, person, and background from the demonstration video was examined. One sample *t*-tests were used to compare the proportion of time spent fixating on the “novel” image to a chance level of looking (.05). For the control group data, one sample *t*-tests were conducted to establish whether the visual recognition image pairs were equally attractive when both were novel for each age group. For the experimental group data, one sample *t*-tests were conducted to assess whether participants showed evidence of recognition memory for the “familiar” image in each pair by showing a visual preference for looking at either the familiar or novel image. As shown in Table 1, with one exception, participants in the control group showed no preference for either image in each visual recognition image pair. Similarly, with two exceptions, participants in the experimental group showed limited evidence of recognition memory for the person, puppet or background from the video. The 12-month-old infants exhibited a preference for the image of the “familiar” background compared to the “novel” background in both the experimental and control groups. This finding suggests a spontaneous preference for the image of the “familiar” background at 12 months of age. In the experimental group, the 9-month-old infants exhibited a preference for the “familiar” person, suggesting recognition for the person at this age.

To determine whether attention during the experimental video was associated with subsequent proportion of looking at the “novel” stimulus during the visual recognition test, correlation analyses were conducted at each age. For the 9-month-old infants there was a positive correlation between attention to the person AOI during learning and the proportion of looking at the “novel” person during the recognition test, *r* = .54, *p* = .048. In other words, increasing attention at the person during the experimental video was related to increasing looking at the “novel” person during the visual recognition test at 9 months of age. There were no other correlations between attention to each AOI and the proportion of looking during the visual recognition test at each age.

### Imitation

Infant recall memory was determined by comparing spontaneous production of the target actions by infants in the control group with target action production by infants in the experimental group. A two-way (Age × Condition) between participants ANOVA was conducted on the imitation scores. Overall, imitation scores did not differ across age, *F*(2, 81) = .98, *p* *=* .379, η^2^ = .15 or condition, *F*(1, 81) = .02, *p* = .889, η^2^ = .01 and there was no interaction *F*(2, 81) = .30, *p* = .741, η^2^ = .08 (see Tab. 2). Thus, when examined separately by age group, infants in the experimental group failed to show evidence of recall for the target actions shown on the video. Moreover, there was no evidence for age-related differences in imitation performance by infants.

The failure to find evidence of infant imitation recall memory can be attributed to the fact that, across age, just 14 out of the 44 infants who completed the imitation test reproduced at least one target action (see Tab. 2). However, the differences in imitation learning outcome between infants might be related to differences in attention during the imitation demonstration video. Thus, for the next analysis, we examined whether there were attentional differences during learning between the “imitators” (*n* = 14) who showed evidence of behavioral recall and the “non imitators” (*n* = 30) who did not. Overall, dwell time to the experimental video did not differ between the “imitators” (*M* = 25.48, *SD* = 1.67) and “non imitators” (*M* = 22.14, *SD* = 1.27, *t*(42) = .74, *p* = .467, *r* = .11). A two-way ANOVA across Imitation recall group (imitators/non-imitators) and AOI revealed a significant interaction between attention to the person, puppet, and background in the video and whether infants imitated the target actions or not, *F*(2, 84) = 4.71, *p* *=* .012, η^2^ = .28. Post hoc *t-*tests revealed that “imitators” looked significantly longer at the person (*M* = 59.18%, *SE* = 7.35) than “non imitators” (*M* = 36.35%, *SE* = 3.93, *t*(42) = 2.60, *p* = .013, *r* = .37) and spent significantly less time looking at the background (*M* = 6.97%, *SE* = 1.72) compared to “non imitators” (*M* = 19.88%, *SE* = 3.36, *t*(42) = −2.54, *p* = .015, *r* = .36). There was no difference between the proportion of time spent attending to the puppet by infants who imitated (*M* = 33.85%, *SE* = 4.24) and those who did not (*M* = 43.77%, *SE* = 7.41, *t*(42) = −1.24, *p* = .221, *r* = .19). Thus, the overall amount of looking to the video was less important for learning than the timing and focus of that looking.

## Discussion

During a video of an imitation demonstration, infants aged 6, 9, and 12 months attended primarily to the target stimulus and the person demonstrating the target actions, but did not preferentially attend to the stimulus. Thus, increasing attention to the puppet, per se, in this imitation task is unlikely to be the central factor in the development of retention and memory flexibility during the infancy period. This finding replicates the findings of Taylor and Herbert (2013) and extends the conclusion that there are no age-related changes in attentional focus in this deferred imitation task throughout the first year of life. Importantly, the present study did, however, reveal significant differences in looking behavior between infants and adults. Overall, adults attended to the video for significantly longer and spent a greater proportion of time attending to the puppet and less time attending to the background compared to the infants. As such, unlike infants, adults’ attentional focus during the imitation demonstration is hierarchical in nature with greater focus on the target stimulus and less focus on peripheral cues (see also, Hard et al., 2011). To some extent changes in the focus of attention between infancy and adulthood may be attributed to infants’ greater distractibility (e.g., Amso & Johnson, 2008). Nevertheless, such differences are likely to influence the way that the same event is encoded into a memory representation (for similar argument, see Jones & Herbert, 2006; Taylor & Herbert, 2013).

The developing ability to focus in on individual components of the video, rather than viewing the scene more holistically (see also Frank et al., 2009), is likely to be a critical factor in the subsequent recall for the target actions presented within the event. In the present study, we found differences in overall patterns of attention between infants who showed evidence of behavioral recall and infants who did not. Infants who imitated the target actions spent a greater proportion of time attending to the person, and less time attending to the background, than infants who did not imitate the actions. Thus, differences in attentional focus are related to subsequent recall for that event. For example, by decreasing attention to the background the importance with which it is encoded in the memory representation may also be decreased, thereby enabling infants to exhibit flexible recall across the physical context change (e.g., a polka dot background in the video and a plain background in the testing room) and 2D (video) to 3D context change (see Barr, 2010). Thus, although there is no age-related change in visual attention during learning, differences in attentional focus are related to differences in encoding and learning outcome within the imitation paradigm by potentially influencing the encoded memory representation (also see Jankowski et al., 2001; Bronson, 1991).

Infants who imitated the target actions also increased their focus of attention to the person rather than to the puppet. Within the imitation literature, successful learning has been linked to interest in the person’s face (Carpenter et al., 1995; Vivanti et al., 2008; Williams et al., 2004) because infants begin to infer the goals of another’s actions (see Behne, Carpenter, Call, & Tomasello, 2005) using facial cues such as facial expression and gaze direction and timing (see Carpenter & Call, 2007). Indeed, young children tend to imitate goals of actions rather than the behavioral means (e.g., Carpenter, Call, & Tomasello, 2005; Gleissner, Bekkering, &, 2000; Loucks & Meltzoff, 2013; Meltzoff, 1995). Adults, in contrast, primarily focused on the puppet in the present study, which may reflect their ability to identify the goals of another’s actions from facial cues quicker than infants. Thus, increased interest in the person by the infants who imitated the target actions might have facilitated their ability to infer the goal of the actions and thus their memory representation for the action demonstrations.

Given that prior studies have found changes in visual attention during the first year of life, the absence of age-related changes in attention between 6 and 12 months in the present study is striking. Age-related changes have previously been documented in studies that presented infants with complex scenes with multiple talking and socially interacting characters (Frank et al., 2009), or when presenting distracters in the visual field (Amso & Johnson, 2008). However, in imitation demonstration videos, the scene is kept relatively simple with a single person presenting actions with one object in front of a background (see Barr et al., 2007). Moreover, the demonstrator in the present study did not speak, and movement was restricted to the action demonstrations only. It is plausible that looking patterns differ according to the complexity of the visual scene being observed. For example, one mechanism by which language cues may enhance learning and memory during an imitation task (e.g., Herbert, 2011) is that the addition of language cues may redirect attentional focus to people and faces. Therefore, age-related changes in attentional focus during the infancy period may be more apparent when observing an imitation demonstration video that includes language cues.

In the present study, attention to the imitation demonstration video changed according to the type of action demonstration being observed. All participants attended longer to the “Shake” action compared to the “Off” and “On” actions. There are three plausible explanations for this finding. First, the sound of the jingle bell during the “Shake” action may have attracted participants’ attention (see Barr, Wyss, & Somander, 2009). However, it is important to note that the jingle bell in the mitten also sounded when the demonstrator tapped the mitten before removing it during the “Off” action. Therefore, we believe this “response to sound” account cannot sufficiently explain the change in attention. Second, the combination of sound and increased movement during the “Shake” action may have attracted attention or equally, the actions performed when the demonstrator looks into the camera (e.g., “Shake”) may be more interesting than actions being performed when the demonstrator looks at the object (e.g., “Off”). Third, the social component driven by the demonstrator’s point of gaze, which was primarily directed at the camera during the “Shake” action compared with the “Off” and “On” actions, may have attracted attention. From 6 months of age, infants can follow an adult’s gaze direction (D’Entremont, Hains, & Muir, 1997) even when presented on a video (Gredebäck, Theuring, Hauf, & Kenward, 2008). Thus, it is plausible that participants are more likely to look longer at the screen when maintaining eye contact with the demonstrator than when following the demonstrator’s gaze to an object.

In support of the “social responsiveness” explanation, participants’ attentional focus increased toward the person during the “Shake” action, but to the puppet during the “Off” and “On” actions. Therefore, it is possible that the increased social component during the “Shake” action may have attracted participants’ attention. This social explanation for attentional focus is consistent with Vivanti et al. (2008), who found that older children increased attention to the person during non-meaningful gestures when the demonstrator looked into the camera compared to meaningful actions with an object when the demonstrator looked at the object. Thus, there are important changes in looking patterns during the learning session which may affect what is encoded and how it is remembered. Indeed, future work could manipulate the social aspects of the imitation task to consider the impact on attentional focus and subsequent memory outcome. Attention to the person may be particularly important for infant learning and memory of the target actions but may be less important for adults who are adept at inferring another’s action goals.

Within the imitation literature, developmental changes in learning and memory have been widely documented (for review, see Hayne, 2004). The level of imitation in the present study was lower than expected given previous research showing that infants can imitate actions demonstrated on a video from 6-months of age (Barr et al., 2007). For successful imitation in the present study, infants had to retrieve their memories across both a salient physical context change (e.g., polka dot background to a plain background) and 2D–3D context change. Typically, when learning from 2D videotaped demonstrations, 6- to 24-month-old infants observe a different context on the video to the one in which they view the video and are unaffected by this context change (e.g., Barr et al., 2007; Strouse & Troseth, 2008). However, due to the salient context used in the present study it is possible that the combined physical and 2D–3D context changes made the task too demanding for infants’ developing flexible memory retrieval abilities (e.g., Hayne et al., 2000; Klein & Meltzoff, 1999; Learmonth et al., 2004). Moreover, prior work demonstrates that small changes to the puppet imitation demonstration video such as the addition of unrelated background music can disrupt infant learning (Barr, Shuck, Salerno, Atkinson, & Linebarger, 2010). Therefore, infant imitation learning in the present experiment was likely disrupted by the use of a salient context on the video.

Although some features of our demonstration video may have reduced infant learning, such as the salient background and the embedding of the video in amongst other events (calibration and a visual recognition test), these features are not dissimilar to what infants would experience when watching infant-directed television programs. Thus, the low level of learning in the present study compared to prior imitation demonstration studies, potentially more closely reflects infant learning from television in everyday life. Importantly, the role of the background as a potential distracter when learning from video has implications for both the imitation literature and infant media.

Overall, there was little evidence of recognition memory for the person, puppet, and background from the demonstration video, with only 9-month-old infants showing recognition of the person (also see Taylor & Herbert, 2013). A number of factors may have resulted in a failure to exhibit recognition memory for components of the demonstration video, and to some extent these factors may differ as a function of age. For the infant participants, recognition failure may have reflected the use of dynamic stimuli at familiarization but static stimuli at test (also see Brown et al., 2006) or the context change between familiarization (polka dot background) and test (plain gray background; e.g., Robinson & Pascalis, 2004). Thus, the mismatch between the cues present at encoding and retrieval may have lead to recognition failure by the infant participants (for review, see Hayne, 2004). For the adult participants, the use of relatively simple stimuli might not have been appropriate to capture evidence of recognition memory. Typically adults are presented with more complex stimuli (e.g., morphing faces, Richmond et al., 2007; 24 pairs of images, McKee & Squire, 1993) for short familiarization times (5–15 s) to account for their faster encoding abilities. Consequently, any evidence of recognition by a novelty preference may have been limited to the first couple of looks since adults may have quickly habituated to the novel image. As such, future work should consider adapting the VPC task for each age group. Nevertheless, recognition memory was related to attention during the imitation demonstration when infants did show evidence of recognition memory at 9 months of age.

In sum, the present study found that differences in attention are related to memory outcome during the first year of infancy. This finding lends support to the argument that changes in attention may influence how an event is encoded in a memory representation and what is available for subsequent recall (also see Jones & Herbert, 2006; Taylor & Herbert, 2013). One of the limitations in the present study is the inability to investigate the role of more fine grained attentional focus on memory outcome. Our ongoing research in this area is investigating whether attention to the puppet’s mitten, puppet’s face and demonstrator’s face change across age and how this might relate to imitation recall memory. We believe it is also important to consider the role of other attentional factors, such as gaze shifts rather than simply attentional focus, on developmental changes in learning and memory. For example, one key area will be to determine how gaze shifts change from anticipatory to predictive across age (see Cannon et al., 2012; Gredebäck et al., 2009) and their relationship to learning outcome. Nevertheless at present, it appears that differences in attention may be one mechanism by which manipulating the amount of learning or cues available during an imitation learning event may enhance infant recall memory (Barr et al., 1996,2003; Barr, Vieira, & Rovee-Collier, 2001; Herbert, 2011).

## Notes

This research was supported by an ESRC studentship (ES/1018786/1) to the first author. The authors would like to thank Harlene Hayne, Jackie Clearwater for providing the imitation stimuli, all the infants, parents who participated in this project.
